# Within-Person Longitudinal Associations Between Obesity-Related Eating Behaviors and Physical Activity Among Older Adults: A Three-Wave Random-Intercept Cross-Lagged Study

**DOI:** 10.3390/healthcare14172747

**Published:** 2026-08-28

**Authors:** Shi Luo, Bin Chen, Xianxiong Li, Joston Gary

**Affiliations:** 1 Faculty of Sports and Exercise Science, University of Malaya, Kuala Lumpur 50603, Malaysia; 24057034@siswa.um.edu.my; 2 College of Physical Education, Jiangxi Normal University, Nanchang 330022, China; 202341900003@jxnu.edu.cn; 3 College of Physical Education, Hunan Normal University, Changsha 410012, China; lixianxiong@hunnu.edu.cn; 4 Department of Management and Engineering, Linköping University, SE-581 83 Linköping, Sweden; 5 Department of Economics, Management, Industrial Engineering and Tourism, University of Aveiro, 3810-193 Aveiro, Portugal; 6 School of Economics, Management and Political Science, University of Minho, 4710-057 Braga, Portugal

**Keywords:** older adults, obesity-related eating behaviors, physical activity, random-intercept cross-lagged panel model, healthy aging

## Abstract

**Objectives**: This study examined the within-person longitudinal associations between obesity-related eating behaviors and physical activity among community-dwelling Chinese older adults. **Methods**: Three waves of data were collected at three-month intervals from 1256 adults aged 65 years or older across 17 urban and rural communities. Physical activity was measured using the Physical Activity Rating Scale-3, and obesity-related eating behaviors were assessed using the seven-item Sakata Eating Behavior Scale Short Form. The eating-behavior scale was reverse-scored so that higher scores represented fewer problematic behaviors. The analysis examined longitudinal measurement invariance, gender differences, and prospective within-person associations using a random-intercept cross-lagged panel model. **Results**: The analysis identified small reciprocal within-person associations between obesity-related eating behaviors and physical activity. Occasions characterized by fewer problematic eating behaviors than usual were followed by higher-than-usual physical activity at the next wave. Higher-than-usual physical activity was likewise followed by fewer problematic eating behaviors. These associations remained similar after adjustment for the available demographic covariates. Men reported higher average physical activity, while women reported fewer problematic eating behaviors, but changes over time did not differ by gender. **Conclusions**: Physical activity and obesity-related eating behaviors showed small bidirectional within-person associations across three-month intervals. This reciprocal pattern suggests that the two behaviors are modestly connected within everyday routines and supports examining them together when studying health behavior dynamics in later life.

## 1. Introduction

Population aging has increased the importance of identifying modifiable behaviors that may help older adults maintain health and functional independence [1,2]. The global population aged 65 years or older is projected to increase from 761 million in 2021 to approximately 1.6 billion by 2050 [3,4]. Healthy aging involves more than preventing disease; it also depends on maintaining mobility, psychological well-being, and participation in daily life [5,6]. Physical activity (PA) and eating behavior are relevant to these outcomes because both form part of everyday routines and may change throughout later life [7,8,9,10].

Eating behavior in later life is shaped by physiological, psychological, and social circumstances. Changes in appetite and taste may interact with health conditions, medication use, oral health, mobility, financial resources, and social isolation [11,12,13,14]. These conditions can affect when older adults eat, how they respond to satiety, and the extent to which eating is prompted by internal needs or external cues. Obesity-related eating behaviors are therefore relevant to weight management and to the ways in which older adults organize and regulate eating in daily life [15,16].

PA represents another behavioral component of health in later life [17,18]. Regular activity supports cardiovascular and metabolic health, muscle strength, balance, mobility, and cognitive functioning [19,20,21,22,23,24,25]. Physical inactivity nevertheless remains widespread. Approximately 31% of adults worldwide did not meet recommended levels of aerobic PA in 2022, with insufficient activity generally becoming more common with age [26]. Declining health, fear of falling, limited access to appropriate facilities, and reduced social support may make it difficult for older adults to remain active [27]. PA may consequently fluctuate as health conditions and daily routines change.

Daily routines provide one possible connection between obesity-related eating behaviors and subsequent PA. Food purchasing and meal preparation require mobility and may connect eating with household responsibilities and social participation [28,29]. Regularly organized eating may also create a predictable structure around which other daily activities can be planned. Associations between eating and activity have been reported in research on dietary patterns, physical functioning, exercise-related eating, and related constructs across different populations [30,31,32,33,34]. The longitudinal issue is whether eating and activity are connected within the same individual over time. A three-wave study of female university students identified prospective associations between restrained eating and PA in both directions [35]. Its findings suggest that temporal links are possible, but the study was conducted among younger participants and examined a different eating-related construct.

A prospective association may also operate from PA to later obesity-related eating behaviors. Exercise can affect appetite control and energy intake, although responses vary across individuals and study settings [36,37]. Regular activity may establish daily structure and provide repeated experiences of successfully managing a health behavior [38,39]. Such experiences could strengthen confidence in managing other daily routines, including eating-related decisions. Psychological well-being provides another possible connection. PA is associated with a lower risk of depression [40], whereas stress and negative emotions can accompany changes in eating behavior [41,42,43].

Social cognitive theory and the Compensatory Carry-Over Action Model provide a framework for considering these processes [44,45]. Social cognitive theory explains behavior through reciprocal relations among personal factors, behavior, and the surrounding environment. Successful regulation in one behavioral domain may strengthen self-efficacy, planning, and perceived control that can later support another behavior. The carry-over model focuses more directly on connections between different health behaviors. It proposes that behaviors may be linked through shared goals and self-regulatory resources. Applied to this study, a temporary deviation from an individual’s usual level of PA or obesity-related eating behaviors could be followed by a later deviation in the other behavior.

These behaviors may develop differently in older women and men. Gender is associated with food preparation roles, opportunities for activity, social resources, health beliefs, and physical limitations in later life [46,47,48,49,50,51]. These circumstances may contribute to differences in average levels of PA and obesity-related eating behaviors over time. These factors do not support a clear directional prediction for gender differences in the longitudinal patterns.

Answering this question requires separating stable differences between older adults from temporary fluctuations within the same individual. Related cross-sectional analyses describe associations between people [52,53,54], while the available longitudinal analysis concerns a younger population and a different eating-related construct [35]. Conventional cross-lagged panel models combine stable between-person differences with within-person fluctuations [55,56]. The random-intercept cross-lagged panel model (RI-CLPM) separates these sources of variation [57,58]. This separation allows the analysis to test whether a deviation from an older adult’s usual level in one behavior predicts a later deviation in the other.

Using three waves of data from community-dwelling Chinese older adults, this study examined whether within-person deviations in obesity-related eating behaviors and PA predicted one another across adjacent waves. The analysis also assessed mean changes in both behaviors and whether those changes differed by gender. An RI-CLPM separated prospective within-person associations from stable between-person differences. The first hypothesis proposed that occasions characterized by fewer problematic obesity-related eating behaviors than usual would predict higher-than-usual PA at the next wave. The reciprocal hypothesis proposed that higher-than-usual PA would predict fewer problematic obesity-related eating behaviors at the next wave. No directional hypothesis was specified for gender differences in mean trajectories.

## 2. Materials and Methods

### 2.1. Participants and Procedure

An a priori power analysis was conducted using G*Power, version 3.1.9.7 (Heinrich Heine University Düsseldorf, Düsseldorf, Germany), for the secondary gender-by-time repeated-measures analysis. Assuming a medium effect size (Cohen’s f = 0.25), α = 0.05, power = 0.95, two gender groups, and three measurement occasions, the minimum required sample size was 251. The final analytic sample included 1256 participants. An achieved-sample Monte Carlo sensitivity assessment was also conducted for the RI-CLPM using the powRICLPM package, version 0.2.1 [58]. Based on 1000 replications, the fitted lagged coefficients, random-intercept correlations, and intraclass correlations of 0.27–0.32, estimated power was approximately 0.70–0.71 for a standardized cross-lagged effect of 0.095 and 0.91–0.93 for an effect of 0.118 (Table S3).

The study used a three-wave longitudinal design. Surveys were completed in May 2025 (T1), August 2025 (T2), and November 2025 (T3). The three-month interval provided two equally spaced observation periods while maintaining the feasibility of repeated community follow-up. Participants came from 17 communities in Hunan, Jiangxi, and Guizhou Provinces. Communities were grouped by province and urban or rural setting. Within these groups, sites were selected according to administrative approval and the feasibility of repeated data collection. Because no probability sampling frame was used and community staff assisted with participant recruitment, the sample covered several regional and residential contexts but was not nationally representative.

Participants were eligible if they (1) were aged 65 years or older; (2) had resided in the selected community for at least 6 months; (3) could understand and complete the questionnaire independently or with standardized assistance from a trained researcher; and (4) provided informed consent. Individuals were excluded if severe cognitive impairment, psychiatric illness, communication difficulties, or serious visual or hearing limitations prevented them from understanding or completing the survey. Questionnaires were also excluded if they contained substantial missing data, evident patterned responding, or internal inconsistencies; were duplicates; or could not be matched across the three waves.

Data were collected using a combination of paper questionnaires and online surveys. Participants familiar with smartphones completed the online version, whereas those who preferred it or required assistance completed a paper questionnaire. The wording, item order, response options, and instructions were identical across the two formats. Trained research assistants administered the surveys using standardized procedures. When participants had difficulty reading an item, the researcher read it aloud without explaining its meaning or suggesting a response. A unique identification code was assigned to each participant to facilitate cross-wave matching while protecting personal information.

**Figure 1:**
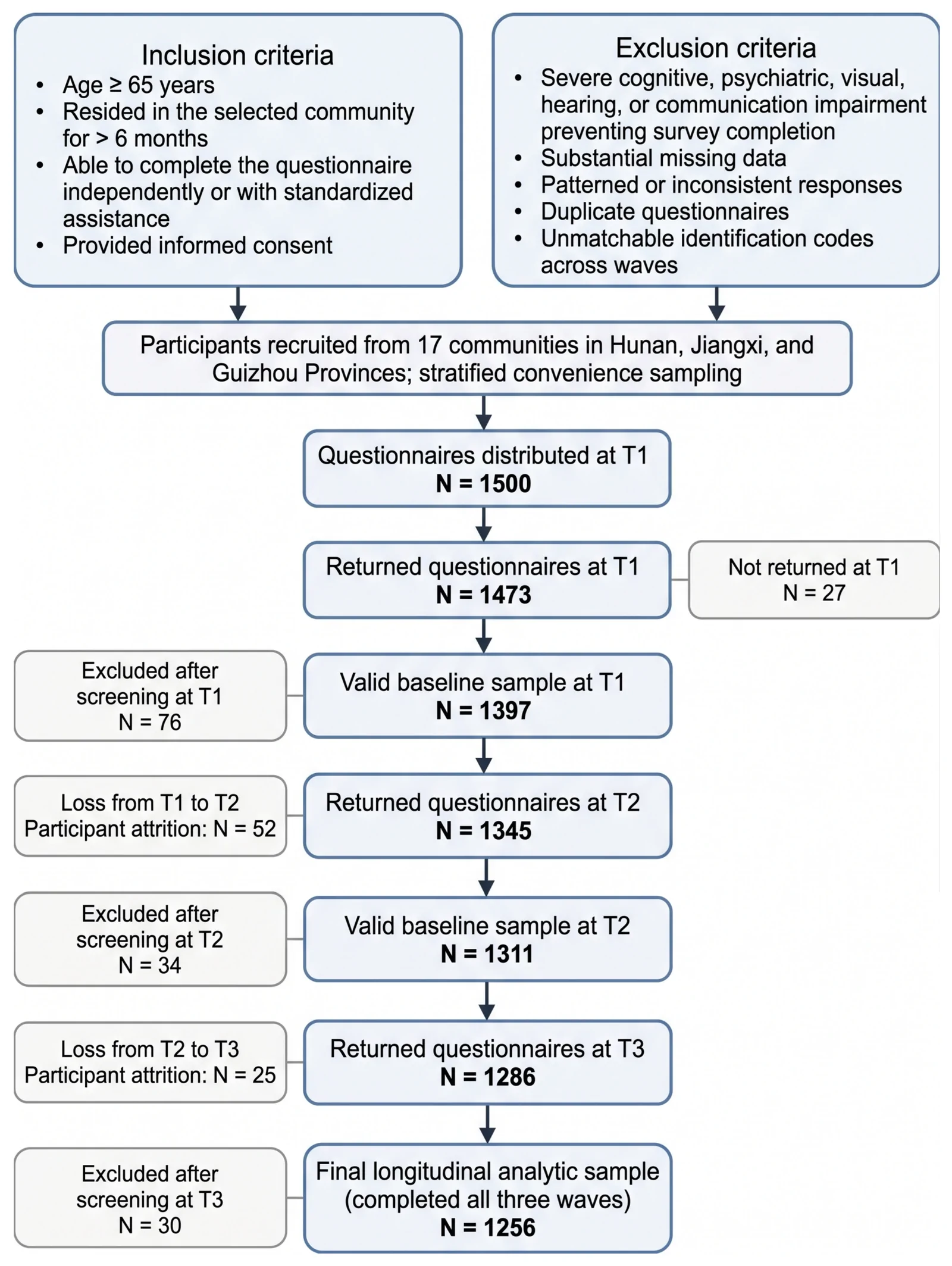
Participant flow through the three-wave longitudinal study.

At T1, 1500 questionnaires were distributed, of which 1473 were returned. After data quality screening, 1397 questionnaires were retained as valid baseline cases, representing 93.1% of all distributed questionnaires. At T2, questionnaires were distributed to the 1397 participants with valid baseline data. A total of 1345 questionnaires were returned, of which 1311 were valid. At T3, questionnaires were distributed to the 1311 participants retained at T2. Of the 1286 returned questionnaires, 1256 met the inclusion and data quality criteria. The final longitudinal analytic sample therefore consisted of 1256 older adults who provided valid data across all three waves. The overall attrition rate from the valid baseline sample to the final assessment was 10.1% (Figure 1).

The final sample had a mean age of 69.00 years (*SD* = 4.96) and included 578 men (46.0%) and 678 women (54.0%). Of the participants, 548 (43.6%) lived in rural areas, and 708 (56.4%) lived in urban areas. The sample included 545 participants (43.4%) from Hunan Province, 346 (27.5%) from Guizhou Province, and 365 (29.1%) from Jiangxi Province. Regarding educational attainment, 449 participants (35.7%) had a primary school education or lower, 456 (36.3%) had completed junior high school, 257 (20.5%) had completed high school or technical secondary school, and 94 (7.5%) had attained a college education or higher. Monthly income was ¥2000 or less for 399 participants (31.8%), ¥2001–3000 for 440 (35.0%), ¥3001–4000 for 236 (18.8%), and more than ¥4000 for 181 (14.4%) (Table 1).

### 2.2. Ethical Considerations

Before each assessment, participants were informed of the study purpose, procedures, confidentiality protections, voluntary nature of participation, and right to withdraw without penalty. Participation proceeded only after written consent had been provided. Ethical approval was obtained before data collection, and all procedures were conducted in accordance with the Declaration of Helsinki. 

### 2.3. Measures

#### 2.3.1. Physical Activity

Physical activity was assessed using the Physical Activity Rating Scale-3 (PARS-3), revised by Liang [59]. The scale assesses PA over the past month using three items addressing exercise intensity, duration, and frequency. Each item uses five ordered response categories. The total PA score is calculated as intensity × (duration − 1) × frequency, yielding a possible score of 0–100. Higher scores indicate greater PA participation. Following the established scoring criteria, scores of 19 or lower represent low PA, scores of 20–42 represent moderate PA, and scores of 43 or higher represent high PA.

#### 2.3.2. Obesity-Related Eating Behaviors

Obesity-related eating behaviors were assessed with the validated Chinese version of the seven-item Sakata Eating Behavior Scale Short Form (EBS-SF). Tayama et al. developed the short form from the original scale using item response theory [15]. Ge et al. translated and validated it in a Chinese population [16]. The items cover eating rhythm, satiety, eating style, perceptions of body constitution, meal content, substitute eating and drinking, and motivation for eating. Each item uses a four-point response scale from 1 (strongly disagree) to 4 (strongly agree). Higher original scores indicate more problematic obesity-related eating behaviors. All items were reverse-scored before being summed, resulting in a total score ranging from 7 to 28, with higher values indicating fewer problematic behaviors. Reverse scoring changed only the direction of the scale; it did not convert the construct into diet quality. Cronbach’s α was 0.849 at T1, 0.856 at T2, and 0.861 at T3.

### 2.4. Data Analysis

IBM SPSS Statistics, version 26.0 (IBM Corp., Armonk, NY, USA), was used for descriptive statistics, correlations, Harman’s single-factor test, and repeated-measures analyses. Mplus, version 8.3 (Muthén & Muthén, Los Angeles, CA, USA), was used for longitudinal measurement invariance analyses. RI-CLPM analyses were conducted in R, version 4.5.2 (R Foundation for Statistical Computing, Vienna, Austria), using the lavaan package, version 0.6-21. The analytic sample included 1256 participants with complete data across all three waves.

Longitudinal measurement invariance was examined separately for the PA components and EBS-SF items. Indicators were treated as continuous, and the models were estimated using maximum likelihood estimation. Configural, metric, and scalar models were tested sequentially. Residuals of corresponding indicators were allowed to correlate across waves. Corresponding factor loadings were constrained in the metric models, while the scalar models additionally constrained the item intercepts. Model fit was evaluated using χ^2^, CFI, TLI, and SRMR. Invariance was supported when ΔCFI was no greater than 0.010 and ΔSRMR was no greater than 0.030 for metric invariance or 0.010 for scalar invariance [60]. For PARS-3, measurement invariance was assessed at the component level, while all substantive analyses used the observed total score calculated with the established multiplicative formula.

Separate 2 (gender: men and women) × 3 (time: T1, T2, and T3) repeated-measures analyses of variance were conducted for PA and reverse-scored obesity-related eating behavior scores. Time was the within-person factor, and gender was the between-person factor. Mauchly’s test assessed sphericity. Greenhouse–Geisser corrections were applied when the assumption of sphericity was violated.

The RI-CLPM was estimated using robust maximum likelihood estimation and separated each observed score into a stable between-person random intercept and a within-person deviation at each wave [56,57]. The model included autoregressive and reciprocal cross-lagged paths among the within-person components. It also estimated the correlation between the random intercepts, the baseline within-person covariance, and residual covariances at T2 and T3. The random intercepts were specified as orthogonal to the within-person components. Corresponding unstandardized autoregressive and cross-lagged paths were constrained to equality across the two three-month intervals. Model fit was evaluated using robust χ^2^, CFI, TLI, RMSEA with its 90% confidence interval, and SRMR. Parameter estimates were reported as standardized coefficients with 95% confidence intervals, together with within-person R^2^ values. In an adjusted sensitivity model, age, gender, residence, province, education, and monthly income were included as predictors of both random intercepts. A conventional first-order CLPM was also estimated for comparison and included only adjacent-wave paths, without direct T1-to-T3 paths. Model specifications and sensitivity results appear in the Supplementary Materials. All tests were two-sided with α = 0.05.

## 3. Results

### 3.1. Common Method Assessment

The first unrotated factor accounted for 32.64% of the total variance, indicating that no single factor accounted for most of the variance.

### 3.2. Descriptive Statistics

Descriptive statistics are presented in Table 2. Mean PA scores varied only slightly across the three waves (M = 26.65–26.77, SD = 29.23–29.32). Mean reverse-scored obesity-related eating behavior scores also varied little across waves (M = 17.65–17.75, SD = 5.56–5.74). PA scores showed moderate positive skewness, whereas reverse-scored obesity-related eating behavior scores were approximately symmetric.

### 3.3. Measurement Invariance Analysis

Longitudinal measurement invariance was examined across T1, T2, and T3 separately for the three PA components and the seven EBS-SF items. Configural, metric, and scalar models were estimated with the residuals of corresponding indicators correlated across waves. The configural models showed good fit for both sets of indicators (Table 3). Constraining corresponding factor loadings and intercepts produced negligible changes in CFI and SRMR, supporting scalar invariance across waves. For PARS-3, scalar invariance was supported at the component level, while subsequent analyses used the observed total score calculated with the established multiplicative formula.

### 3.4. Correlations Among the Study Variables

Table 4 presents the bivariate correlations among the study variables. PA scores were positively correlated across waves (r = 0.350–0.493), as were reverse-scored obesity-related eating behavior scores (r = 0.418–0.592). Cross-domain correlations between PA and reverse-scored obesity-related eating behavior scores ranged from r = 0.245 to 0.396. All correlations were statistically significant at *p* < 0.01.

### 3.5. Gender Differences in the Study Variables

Mauchly’s test indicated that the assumption of sphericity was violated for PA, W = 0.971, χ^2^(2) = 36.776, *p* < 0.001, and reverse-scored obesity-related eating behavior scores, W = 0.944, χ^2^(2) = 71.841, *p* < 0.001. Greenhouse–Geisser-corrected results were therefore reported. Table 5 summarizes the repeated-measures ANOVA results.

PA did not change significantly over time, F(1.944, 2437.498) = 0.007, *p* = 0.992, and the time-by-gender interaction was also nonsignificant, F(1.944, 2437.498) = 0.108, *p* = 0.893. Men reported higher average PA than women, F(1, 1254) = 50.739, *p* < 0.001.

Reverse-scored obesity-related eating behavior scores did not change significantly over time, F(1.894, 2375.625) = 0.223, *p* = 0.788. The time-by-gender interaction was nonsignificant, F(1.894, 2375.625) = 1.396, *p* = 0.248. Women reported higher average reverse-scored obesity-related eating behavior scores than men, F(1, 1254) = 46.790, *p* < 0.001, indicating fewer problematic obesity-related eating behaviors (Table 5).

### 3.6. Random-Intercept Cross-Lagged Panel Model Results

**Figure 2:**
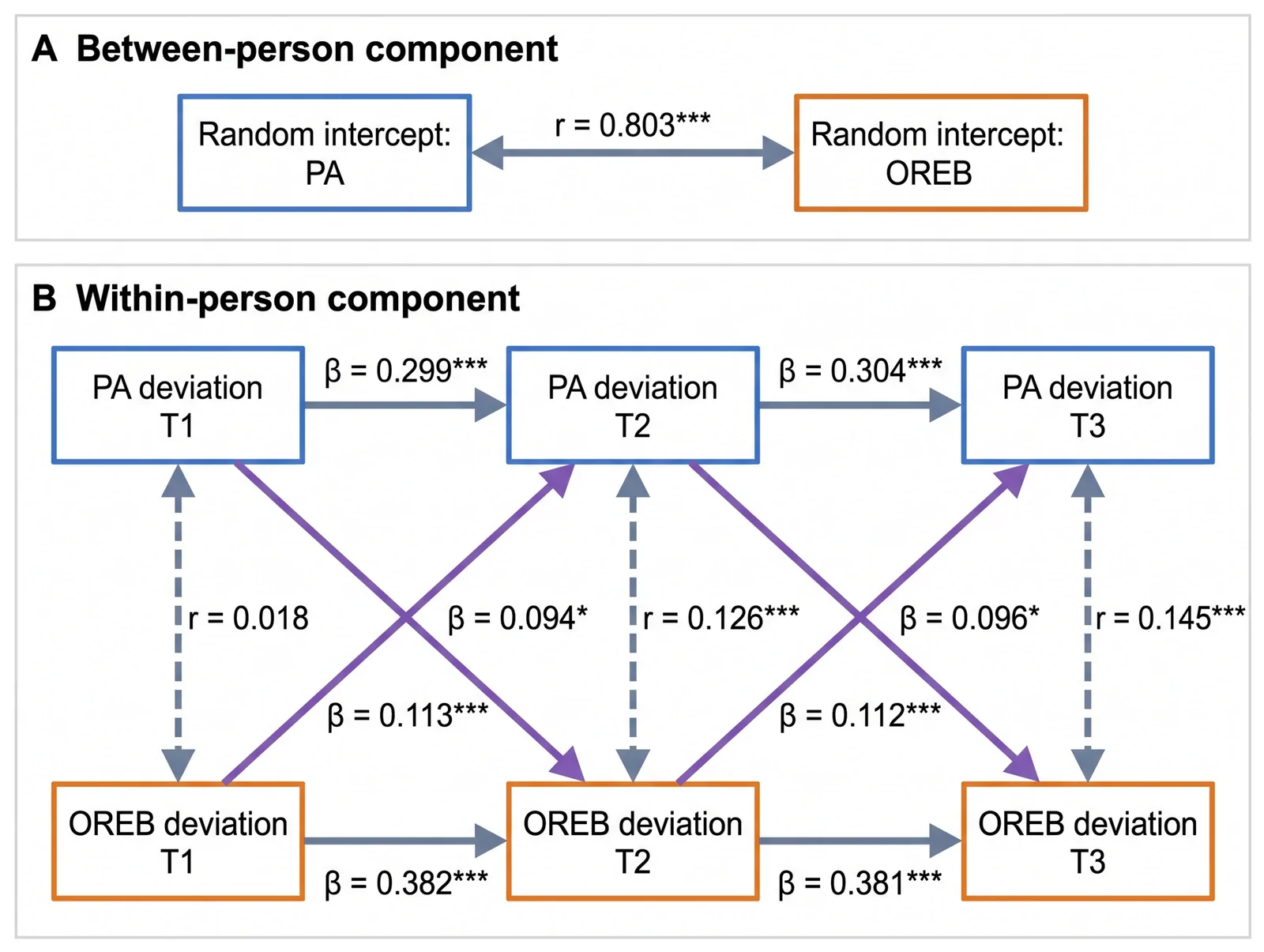
Random-intercept cross-lagged panel model. Note. Standardized coefficients are shown. Within-wave arrows represent the baseline correlation at T1 and residual correlations at T2 and T3. * *p* < 0.05; *** *p* < 0.001.

The RI-CLPM showed good fit to the data: χ^2^(5) = 3.620, *p* = 0.605, CFI = 1.000, TLI = 1.000, RMSEA = 0.000, 90% CI [0.000, 0.034], and SRMR = 0.011. The random-intercept variances were 226.18 for PA, 95% CI [144.20, 308.16], and 9.92 for reverse-scored obesity-related eating behavior scores, 95% CI [6.82, 13.01], both *p* < 0.001. The two random intercepts were strongly positively correlated, r = 0.803, *p* < 0.001 (Figure 2).

All autoregressive and reciprocal cross-lagged paths were positive and statistically significant (Figure 2 and Table 6). Standardized autoregressive coefficients ranged from 0.299 to 0.304 for PA and from 0.381 to 0.382 for reverse-scored obesity-related eating behavior scores. Cross-lagged coefficients ranged from 0.094 to 0.096 from reverse-scored obesity-related eating behavior scores to subsequent PA and from 0.112 to 0.113 from PA to subsequent reverse-scored obesity-related eating behavior scores. At the within-person level, occasions characterized by fewer problematic obesity-related eating behaviors than usual were followed by higher-than-usual PA at the next wave. Higher-than-usual PA was likewise followed by fewer problematic obesity-related eating behaviors at the next wave.

The baseline within-person correlation at T1 was small and nonsignificant, r = 0.018, *p* = 0.739. Residual correlations were positive at T2, r = 0.126, *p* < 0.001, and T3, r = 0.145, *p* < 0.001. Within-person R^2^ values ranged from 0.099 to 0.112 for PA and from 0.160 to 0.173 for reverse-scored obesity-related eating behavior scores. Covariate-adjusted estimates were similar to the primary estimates (Table S2), while the conventional CLPM produced larger cross-lagged estimates (Table S1).

## 4. Discussion

This study identified small reciprocal within-person associations between physical activity (PA) and obesity-related eating behaviors across successive three-month intervals. Occasions characterized by higher reverse-scored obesity-related eating behavior scores than usual, indicating fewer problematic behaviors, were followed by slightly higher-than-usual PA at the next wave. Higher-than-usual PA was likewise followed by higher reverse-scored obesity-related eating behavior scores. The standardized cross-lagged coefficients ranged from 0.094 to 0.113, indicating modest associations. Adjustment for age, gender, residence, province, education, and income produced similar estimates.

The distinction between the between-person and within-person results clarifies the meaning of these associations. The strong correlation between the random intercepts (r = 0.803) showed that participants who were usually more active also tended to report fewer problematic obesity-related eating behaviors across the study period. This correlation represented stable differences between participants. The cross-lagged paths addressed a different question: whether a temporary deviation from a participant’s usual level in one behavior was associated with a subsequent deviation in the other behavior. The conventional CLPM produced larger cross-lagged coefficients because it did not separate stable between-person differences from within-person fluctuations [55]. The comparison shows that the two behaviors were strongly related across individuals but only modestly connected through short-term changes within the same individual.

The estimated within-person paths from reverse-scored obesity-related eating behavior scores to subsequent PA were positive and small at both three-month intervals (β = 0.094 and 0.096). Previous research has connected eating behaviors among older adults with psychosocial conditions, mobility, social participation, and the organization of everyday life [28,29]. Eating behaviors may also remain relatively stable during later adulthood [61]. A temporary shift toward fewer problematic eating behaviors may therefore occur during periods characterized by more regular daily routines or more consistent management of health-related activities. These periods may also provide greater opportunities for walking, household activity, and social participation. Direct measurement of meal routines, daily schedules, and social engagement would help determine whether these processes explain the observed association.

The reciprocal path from PA to subsequent reverse-scored obesity-related eating behavior scores was also positive and small. Exercise research has documented changes in appetite control, satiety sensitivity, and some eating behavior traits across different populations and activity conditions [36,62,63]. Although these studies concern eating-related outcomes that are not identical to the EBS-SF, they indicate that activity may coincide with later changes in eating regulation. PA may also introduce structure into the day and provide repeated experiences of managing a health behavior [64]. Such experiences may support self-regulation in subsequent food-related decisions [65,66,67,68]. Physical functioning, health consciousness, chronic disease, medication use, household resources, and social support may additionally contribute to changes in both behaviors [69,70,71,72,73,74]. The present analysis cannot determine whether physiological appetite regulation, daily structure, self-regulation, or another process accounts for the observed association.

The reciprocal pattern is broadly compatible with social cognitive theory and the Compensatory Carry-Over Action Model [44,45]. Both perspectives describe how experiences, expectations, self-efficacy, shared goals, and regulatory resources may connect behaviors across different domains. The findings place this theoretical expectation in a within-person longitudinal context: a temporary change in one health behavior may precede a later change in another behavior. The modest coefficients indicate that cross-behavior carry-over was limited over the three-month observation interval. Studies using more frequent assessments could measure self-efficacy, behavioral intentions, shared goals, and regulatory resources alongside both behaviors to establish when these processes occur.

Gender differences concerned average behavioral levels and mean trajectories. Men reported higher average PA, whereas women reported higher reverse-scored obesity-related eating behavior scores and thus fewer problematic behaviors. Earlier studies have also identified gender differences in older adults’ activity patterns, health practices, and eating-related experiences [26,46,48,49,50,51,75]. Household responsibilities, food preparation roles, neighborhood opportunities, perceived safety, and physical limitations may contribute to these differences [76,77,78]. The nonsignificant time-by-gender interactions showed that mean changes across the three assessments did not differ detectably between women and men. These analyses characterize gender differences in average levels and short-term mean trajectories. Gender variation in within-person cross-lagged associations remains a empirical question.

### 4.1. Implications

The main methodological implication concerns the separation of stable between-person differences from within-person behavioral change. The strong random-intercept correlation and the much smaller cross-lagged coefficients show that a strong association between older adults does not necessarily imply a similarly strong connection between changes occurring within the same individual. Cross-sectional analyses and longitudinal models that combine these levels may assign stable differences in health, socioeconomic resources, living conditions, or behavioral disposition to short-term behavioral dynamics. Longitudinal research on multiple health behaviors should therefore align the analytical level with the research question. Additional measurement waves and comparisons across different observation intervals would help establish whether the reciprocal associations vary over shorter or longer periods.

The reciprocal associations also support treating PA and obesity-related eating behaviors as related outcomes when evaluating health behavior programs for older adults. Programs primarily addressing one behavior could include the other as a secondary outcome, allowing possible cross-behavior changes to be measured directly. Future randomized trials could compare a combined program with separate programs addressing PA and obesity-related eating behaviors. Such trials could also measure self-efficacy, daily routines, social participation, and appetite regulation to identify the conditions under which change carries across behavioral domains. Evidence on multiple health behavior interventions among older adults remains limited [79]. Direct comparisons would clarify whether combined programs provide additional benefits and which participants are most likely to experience reciprocal behavioral change.

### 4.2. Strengths and Limitations

The study has several strengths. The large sample, three equally spaced assessments, and recruitment across urban and rural communities in three provinces supported the longitudinal analysis. Longitudinal measurement consistency was assessed before the main analyses, and the RI-CLPM separated stable between-person differences from within-person fluctuations. The covariate-adjusted model produced similar estimates, while the conventional CLPM comparison showed how model choice affected the magnitude and interpretation of the cross-lagged estimates.

Several limitations define the scope of the findings. First, the study used non-probability community sampling. Communities were selected according to administrative cooperation and the feasibility of repeated follow-up, and no national probability frame was used. The findings are most applicable to older adults in similar community settings. Second, the analytic sample included 1256 participants with complete data across all three waves. Baseline records for the 141 participants who were not retained through T3 were unavailable, preventing comparisons between completers and non-completers and assessment with Little’s MCAR test. Attrition may consequently have affected the composition of the final sample. 

Third, PA and obesity-related eating behaviors were self-reported. Recall, social desirability, and a shared assessment method may have affected the estimated associations. Future studies could combine device-measured activity with repeated or independent assessments of eating behavior. Fourth, the EBS-SF measures obesity-related eating behaviors and does not represent diet quality, nutrient adequacy, dietary diversity, or malnutrition risk. Its reverse-scored total indicates fewer problematic behaviors within the measured construct. Similarly, PARS-3 is a multiplicative composite. Its measurement invariance results concern the consistency of the intensity, duration, and frequency components, while the main analyses concern the observed total score calculated with the established formula.

Fifth, the available covariates included age, gender, residence, province, education, and income, but the dataset did not include BMI, chronic disease, medication use, functional capacity, or cognitive status. These unmeasured characteristics may contribute to both PA and obesity-related eating behaviors. Sixth, the two three-month intervals represent a single observation scale and may also include seasonal influences. Studies covering additional intervals could determine whether the reciprocal associations differ across shorter periods, longer periods, or seasons.

Seventh, a three-wave RI-CLPM provides a limited representation of within-person behavioral dynamics, and the Monte Carlo sensitivity assessment indicated lower power for the smaller eating-behavior-to-PA path. Replication with more waves and larger longitudinal samples would provide more precise estimates. Finally, the observational design establishes prospective ordering but does not identify causal effects.

## 5. Conclusions

Among community-dwelling Chinese older adults, PA and reverse-scored obesity-related eating behavior scores were connected at both the between-person and within-person levels. Older adults who were generally more active also tended to report fewer problematic obesity-related eating behaviors, while temporary deviations in the two behaviors showed small reciprocal associations across successive three-month intervals. Occasions characterized by fewer problematic eating behaviors than usual preceded higher-than-usual PA, and higher-than-usual PA preceded fewer problematic eating behaviors. These associations remained similar after adjustment for the available demographic covariates.

By separating stable differences between participants from changes occurring within the same participant, the RI-CLPM showed that the two behaviors were strongly related across individuals but only modestly connected through short-term within-person change. These findings support examining PA and obesity-related eating behaviors as related but distinct components of health behavior in later life across multiple measurement intervals.

## Figures and Tables

**Table 1:** Demographic characteristics of the study participants.

Characteristic	Category	Count	N %
Gender	Men	578	46.0%
	Women	678	54.0%
Residence	Rural	548	43.6%
	Urban	708	56.4%
Province	Hunan	545	43.4%
	Guizhou	346	27.5%
	Jiangxi	365	29.1%
Education	Primary school or below	449	35.7%
	Junior high school	456	36.3%
	High school/technical secondary school	257	20.5%
	College or above	94	7.5%
Monthly Income	≤2000	399	31.8%
	2001–3000	440	35.0%
	3001–4000	236	18.8%
	>4000	181	14.4%

**Table 2:** Descriptive statistics for physical activity and reverse-scored obesity-related eating behavior scores across T1–T3.

Variable	Mean	Std. Deviation	Skewness	Kurtosis
T1PA	26.77	29.26	1.195	0.404
T2PA	26.65	29.23	1.197	0.397
T3PA	26.76	29.32	1.188	0.381
T1OREB	17.67	5.56	0.017	−0.968
T2OREB	17.75	5.60	−0.093	−0.987
T3OREB	17.65	5.74	−0.050	−1.022

**Table 3:** Longitudinal measurement invariance of physical activity components and obesity-related eating behavior items across T1–T3.

Construct	Model	χ^2^ (*df*)	*p*	CFI	TLI	SRMR	ΔCFI	ΔSRMR
PA	Configural	14.47(15)	0.49	1	1	0.009	—	—
	Metric	15.04 (19)	0.72	1	1	0.01	0	0.001
	Scalar	17.2 (23)	0.80	1	1	0.011	0	0.001
OREB	Configural	163.39(165)	0.52	1	1	0.017	—	—
	Metric	169.92(177)	0.64	1	1	0.019	0	0.002
	Scalar	182.96(189)	0.61	1	1	0.019	0	0

Note. PA = physical activity; OREB = reverse-scored obesity-related eating behavior score; CFI = comparative fit index; TLI = Tucker–Lewis index; SRMR = standardized root mean square residual.

**Table 4:** Pearson correlations among physical activity and reverse-scored obesity-related eating behavior scores across T1–T3.

Variable	T1PA	T2PA	T3PA	T1OREB	T2OREB	T3OREB
T1PA	1					
T2PA	0.488 **	1				
T3PA	0.350 **	0.493 **	1			
T1OREB	0.245 **	0.274 **	0.277 **	1		
T2OREB	0.313 **	0.345 **	0.358 **	0.570 **	1	
T3OREB	0.295 **	0.355 **	0.396 **	0.418 **	0.592 **	1

Note. PA = physical activity; OREB = reverse-scored obesity-related eating behavior score. ** *p* < 0.01.

**Table 5:** Repeated-measures ANOVA results for physical activity and reverse-scored obesity-related eating behavior scores.

Outcome	Effect	*F*	*df*	*p*
PA	Time	0.007	1.944, 2437.498	0.992
	Gender	50.739	1, 1254	<0.001
	Time × gender	0.108	1.944, 2437.498	0.893
OREB	Time	0.223	1.894, 2375.625	0.788
	Gender	46.790	1, 1254	<0.001
	Time × gender	1.396	1.894, 2375.625	0.248

Note. Greenhouse–Geisser-corrected degrees of freedom are reported for time and time × gender effects.

**Table 6:** Standardized within-person estimates in the random-intercept cross-lagged panel model.

Parameter	*β* or *r*	95% CI	*p*	Outcome R^2^
PA T1 -> PA T2	0.299	0.206, 0.392	<0.001	0.099
OREB T1 -> PA T2	0.094	0.022, 0.165	0.010	
OREB T1 -> OREB T2	0.382	0.299, 0.466	<0.001	0.160
PA T1 -> OREB T2	0.113	0.046, 0.180	<0.001	
PA T2 -> PA T3	0.304	0.208, 0.401	<0.001	0.112
OREB T2 -> PA T3	0.096	0.022, 0.170	0.011	
OREB T2 -> OREB T3	0.381	0.292, 0.470	<0.001	0.173
PA T2 -> OREB T3	0.112	0.046, 0.179	<0.001	
T1 correlation	0.018	−0.089, 0.126	0.739	
T2 residual correlation	0.126	0.052, 0.200	<0.001	
T3 residual correlation	0.145	0.077, 0.213	<0.001	

Note. OREB = reverse-scored obesity-related eating behavior score. T1 reports the baseline within-person correlation; T2 and T3 report residual correlations.

## Data Availability

The data supporting the findings of this study are available from the corresponding author upon reasonable request. The data are not publicly available due to participant privacy considerations and restrictions associated with the informed consent provided by the participants.

## Supplementary Materials

The following supporting information can be downloaded at https://www.mdpi.com/article/10.3390/healthcare14172747/s1, Supplementary Methods S1: RI-CLPM specification and stationarity assessment; Table S1: Conventional cross-lagged panel model sensitivity analysis; Table S2: RI-CLPM sensitivity analysis adjusted for available demographic covariates; Table S3: Achieved-sample Monte Carlo sensitivity assessment for RI-CLPM cross-lagged paths.

## Abbreviations

The following abbreviations are used in this manuscript:

**Abbreviation**	**Definition**
PA	Physical activity
**OREB**	Obesity-related eating behavior score
CFI	Comparative fit index
CLPM	Cross-lagged panel model
EBS-SF	Sakata Eating Behavior Scale Short Form
MLR	Robust maximum likelihood
PARS-3	Physical Activity Rating Scale-3
RMSEA	Root mean square error of approximation
SRMR	Standardized root mean square residual
TLI	Tucker–Lewis index
**RI-CLPM**	Random-intercept cross-lagged panel model
