# Supplementary material for: Within-Person Longitudinal Associations Between Obesity-Related Eating Behaviors and Physical Activity Among Older Adults: A Three-Wave Random-Intercept Cross-Lagged Study
Source: Healthcare (Basel). 2026 Aug 28;14(17):2747. doi: 10.3390/healthcare14172747 (PMC13565216; doi:10.3390/healthcare14172747)
Supplement: Supplementary file 1 [file healthcare-14-02747-s001.zip › Supplementary Methods S1.pdf]

Within-Person Longitudinal Associations Between Obesity-Related Eating Behaviors and Physical Activity Among Older Adults: A Three-Wave Random-Intercept Cross-Lagged Study

Supplementary Materials

Supplementary Methods S1. RI-CLPM specification and stationarity assessment

The main RI-CLPM used six observed total scores and was estimated with robust maximum likelihood (MLR). Random-intercept factors captured stable between-person differences, while single-indicator latent variables represented within-person deviations. Observed residual variances were fixed at zero for exact decomposition, and the random intercepts were orthogonal to all within-person components. The model estimated autoregressive and reciprocal cross-lagged paths among the within-person components, the correlation between random intercepts, the baseline within-person covariance, and residual covariances at T2 and T3. Corresponding unstandardized autoregressive and cross-lagged paths were constrained equal across the two three-month intervals. Standardized estimates could differ slightly because variances differed across waves. A fully time-varying lagged model produced an inadmissible solution with a singular information matrix and negative within-person variances. We therefore examined two partial-constraint models. Neither freeing the cross-lagged paths, $p = 0.235$, nor freeing the autoregressive paths, $p = 0.823$, improved fit over the selected model.
