# Supplementary material for: Within-Person Longitudinal Associations Between Obesity-Related Eating Behaviors and Physical Activity Among Older Adults: A Three-Wave Random-Intercept Cross-Lagged Study
Source: Healthcare (Basel). 2026 Aug 28;14(17):2747. doi: 10.3390/healthcare14172747 (PMC13565216; doi:10.3390/healthcare14172747)
Supplement: Supplementary file 1 [file healthcare-14-02747-s001.zip › Table S1.pdf]

Within-Person Longitudinal Associations Between Obesity-Related Eating Behaviors and Physical Activity Among Older Adults: A Three-Wave Random-Intercept Cross-Lagged Study

Supplementary Materials

Table S1. Conventional cross-lagged panel model sensitivity analysis.

**Path**	***β***	**95% CI**	***p***
PA T1 → PA T2	0.448	0.395, 0.501	<0.001
OREB T1 → PA T2	0.164	0.115, 0.213	<0.001
PA T1 → OREB T2	0.185	0.136, 0.234	<0.001
OREB T1 → OREB T2	0.524	0.482, 0.567	<0.001
PA T2 → PA T3	0.420	0.366, 0.474	<0.001
OREB T2 → PA T3	0.213	0.163, 0.262	<0.001
PA T2 → OREB T3	0.171	0.125, 0.217	<0.001
OREB T2 → OREB T3	0.533	0.492, 0.574	<0.001

Note. Robust fit: $\chi^2(4) = 30.001$, $p < 0.001$; CFI = 0.987; TLI = 0.951; RMSEA = 0.074, 90% CI [0.051, 0.101]; SRMR = 0.023. The model contained only adjacent-wave first-order paths. With six observed variables, the covariance structure has 21 distinct moments and 17 free covariance, regression, and variance parameters, yielding 4 degrees of freedom. PA = physical activity; OREB = reverse-scored obesity-related eating behavior score.
