# Supplementary material for: Within-Person Longitudinal Associations Between Obesity-Related Eating Behaviors and Physical Activity Among Older Adults: A Three-Wave Random-Intercept Cross-Lagged Study
Source: Healthcare (Basel). 2026 Aug 28;14(17):2747. doi: 10.3390/healthcare14172747 (PMC13565216; doi:10.3390/healthcare14172747)
Supplement: Supplementary file 1 [file healthcare-14-02747-s001.zip › Table S2.pdf]

Within-Person Longitudinal Associations Between Obesity-Related Eating Behaviors and Physical Activity Among Older Adults: A Three-Wave Random-Intercept Cross-Lagged Study

Supplementary Materials

Table S2. RI-CLPM sensitivity analysis adjusted for available demographic covariates.

Path	β	95% CI	p
PA T1 → PA T2	0.302	0.211, 0.394	<0.001
OREB T1 → PA T2	0.094	0.023, 0.165	0.009
PA T1 → OREB T2	0.119	0.052, 0.186	<0.001
OREB T1 → OREB T2	0.375	0.292, 0.457	<0.001
PA T2 → PA T3	0.307	0.214, 0.401	<0.001
OREB T2 → PA T3	0.095	0.022, 0.168	0.010
PA T2 → OREB T3	0.117	0.051, 0.184	<0.001
OREB T2 → OREB T3	0.368	0.281, 0.455	<0.001

Note. Age, gender, residence, province, education, and monthly income were included as predictors of the PA and OREB random intercepts. Robust fit: $\chi^2(33) = 29.314$, $p = 0.651$; CFI = 1.000; TLI = 1.000; RMSEA = 0.000, 90% CI [0.000, 0.017]; SRMR = 0.012. PA = physical activity; OREB = reverse-scored obesity-related eating behavior score.
