# Supplementary material for: Within-Person Longitudinal Associations Between Obesity-Related Eating Behaviors and Physical Activity Among Older Adults: A Three-Wave Random-Intercept Cross-Lagged Study
Source: Healthcare (Basel). 2026 Aug 28;14(17):2747. doi: 10.3390/healthcare14172747 (PMC13565216; doi:10.3390/healthcare14172747)
Supplement: Supplementary file 1 [file healthcare-14-02747-s001.zip › Table S3.pdf]

Within-Person Longitudinal Associations Between Obesity-Related Eating Behaviors and Physical Activity Among Older Adults: A Three-Wave Random-Intercept Cross-Lagged Study

Supplementary Materials

Table S3. Achieved-sample Monte Carlo sensitivity assessment for RI-CLPM cross-lagged paths.

ICC	Population β	Admissible replications	Estimated power	95% CI coverage
0.27	0.095 (OREB → PA)	894	0.697	0.959
0.27	0.118 (PA → OREB)	894	0.925	0.952
0.32	0.095 (OREB → PA)	956	0.708	0.968
0.32	0.118 (PA → OREB)	956	0.906	0.951

Note. N = 1,256; three waves; 1,000 replications per condition; α = 0.05; MLR estimator; equal lagged paths; random-intercept correlation = 0.80; baseline within-person correlation = 0.02. Population β values were based on the rounded cross-lagged coefficients from the demographic-adjusted RI-CLPM. Power estimates use admissible replications. This was an achieved-sample sensitivity assessment, not an a priori design calculation. ICC = intraclass correlation; MLR = robust maximum likelihood; PA = physical activity; OREB = reverse-scored obesity-related eating behavior score.
